# Structure, dielectric, and piezoelectric properties of K_0.5_Na_0.5_NbO_3_-based lead-free ceramics

**DOI:** 10.1039/c8ra04038a

**Published:** 2018-07-05

**Authors:** Sushmita Dwivedi, Tanvi Pareek, Sunil Kumar

**Affiliations:** Discipline of Metallurgy Engineering and Materials Science, Indian Institute of Technology Indore Simrol 453552 India sunil@iiti.ac.in +91-7324306-685

## Abstract

Lead-free ceramics based on the (1 − *x*)K_0.5_Na_0.5_NbO_3_–*x*Bi(Zn_0.5_Ti_0.5_)O_3_ (KNN–BZT) system obtained *via* the conventional solid-state processing technique were characterized for their crystal structure, microstructure, and electrical properties. Rietveld analysis of X-ray diffraction data confirmed the formation of a stable perovskite phase for Bi(Zn_0.5_Ti_0.5_)O_3_ substitutions up to 30 mol%. The crystal structure was found to transform from orthorhombic *Amm*2 to cubic *Pm*3̄*m* through mixed rhombohedral and tetragonal phases with the increase in Bi(Zn_0.5_Ti_0.5_)O_3_ content. Temperature-dependent dielectric behavior indicated an increase in diffuseness of both orthorhombic to tetragonal and tetragonal to cubic phase transitions as well as a gradual shift towards room temperature. The sample with *x* ≈ 0.02 exhibited a mixed rhombohedral and orthorhombic phase at room temperature. A high-temperature X-ray diffraction study confirmed the strong temperature dependence of the phase coexistence. The sample with the composition 0.98(K_0.5_Na_0.5_NbO_3_)–0.02(BiZn_0.5_Ti_0.5_O_3_) showed an improved room temperature piezoelectric coefficient *d*_33_ = 109 pC/N and a high Curie temperature *T*_C_ = 383 °C.

## Abstract 2

Room temperature powder X-ray diffraction patterns of (1 – *x*)K_0.5_Na_0.5_NbO_3_–*x*Bi(Zn_0.5_Ti_0.5_)O_3_ system.

## Graphical abstract

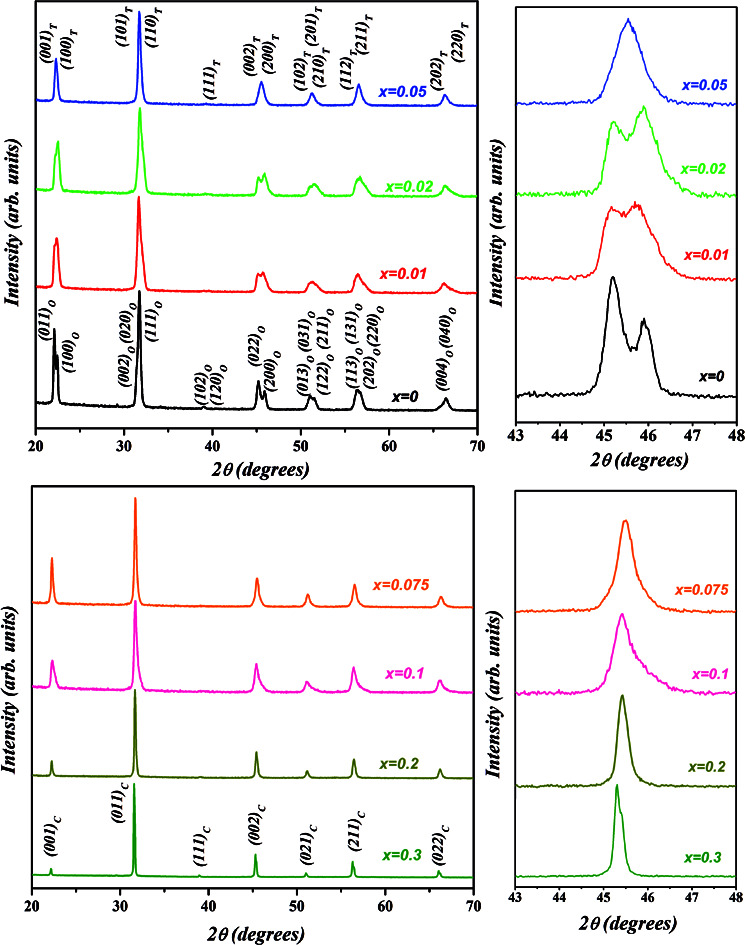

## 1. Introduction

The development of piezoelectric materials has led to an extensive range of applications from regular use to more specialized devices such as sensors, actuators, in medical devices, *etc.*^1,2^ It is well known that perovskite ferroelectric materials possess high piezoelectricity. In this regard, Pb(Zr,Ti)O_3_ (PZT) based materials have been widely used due to their excellent piezoelectric properties, high Curie temperature, large-scale production and the possibility of changing properties through composition.^2,3^ However, lead-based materials are restricted by global regulations due to the high toxicity and irreversible hazards of lead exposure. This has led to tremendous efforts in the compositional development of lead-free systems exhibiting an MPB like that in PZT with improved properties. Currently, three main groups of perovskite materials are being considered: K_0.5_Na_0.5_NbO_3_ (KNN)-based, BaTiO_3_ (BT)-based, and Na_0.5_Bi_0.5_TiO_3_ (NBT)-based piezoelectrics.^1,3–11^ Among these ceramics (K,Na)NbO_3_ based ceramics are promising candidates for replacing PZT because of superior piezoelectric characteristics and a relatively high Curie temperature (*T*_C_).^6,8,10–13^

Recently, a number of manpower and financial resources are invested into the study of KNN-based piezoelectrics, and some significant developments have been achieved.^9–18^ KNN is a solid solution of ferroelectric compound KNbO_3_ having space group *Cm*2*m* and antiferroelectric NaNbO_3_ having space group *Pbma* with an orthorhombic perovskite structure at room temperature.^16^ The composition near *x* = 0.5 is of the great interest because of the superior ferroelectric and piezoelectric properties. K_0.5_Na_0.5_NbO_3_ undergoes three phase transitions: rhombohedral to orthorhombic (*T*_R–O_) around −110 °C, orthorhombic to tetragonal (*T*_O–T_) around 180 °C, and tetragonal to cubic (*T*_T–C_) around 400 °C.^5,6,10^ KNN ceramics show a quite high piezoelectric response, excellent electromechanical coupling coefficient and a relatively low room temperature dielectric constant.^7,10,12,19,20^ The lower theoretical density of KNN ceramics as compared to PZT is another obvious advantage as it helps in designing lighter piezo-active elements for a variety of applications. For transducers applications, lower-density KNN-based ceramics are expected to have lower acoustical impedance. The compatibility of KNN with animal and human tissue in dense ceramics form makes its attractive in the biomedical field.^1^ These properties make KNN potentially viable alternative to lead-based materials for a variety of applications. One of the significant challenges in the development of KNN ceramics for piezoelectric applications is to obtain dense ceramic with good electrical properties using conventional sintering methods due to the high volatility of alkaline elements. Several studies have been carried out to enhance the piezoelectric properties through the modification of the parent KNN compound by substitutions of some cations at A-site and/or B-site of KNN structure and microstructural engineering.^13,18,20–33^ For instance, KNN systems modified by LiTaO_3_ and LiSbO_3_ have improved the densification as well as enhanced the piezoelectric properties (*d*_33_ = 200–235 pC/N).^23,24,34–36^ Saito *et al.* in 2004 reported a peak *d*_33_ of ∼416 pC/N for textured KNN-based ceramics.^5^ A large piezoelectric coefficient *d*_33_ of ∼490 pC/N in KNN-based composition was achieved by Wang *et al.* by introducing the rhombohedral and tetragonal (R–T) phase boundary.^22,37^ Similarly, an ultrahigh piezoelectric coefficient *d*_33_ of ∼700 pC/N and an excellent electromechanical planar coupling coefficient of 76% have been demonstrated in KNN-based textured ceramics by Li *et al.*^38^ With the addition of dopants such as Li, Ta, and Sb, highly-dense samples of KNN have been produced using traditional sintering. In some cases, the dopants are also used to improve the room temperature piezoelectric properties by decreasing the orthorhombic to tetragonal polymorphic phase boundary (PPB) to near room temperature.^6,10,11^ Such improvements in room temperature piezoelectric properties usually comes at the expense of lowered *T*_C_.

Perovskite ferroelectric compounds with A-site occupied by an element with stereochemically active 6s^2^ lone pair are expected to exhibit high *T*_C_ and a large remnant polarization due to the large distortion in crystal structure induced by the A-site.^39^ BiZn_0.5_Ti_0.5_O_3_ (BZT) is a perovskite-structured compound with an extremely high tetragonal strain (*c*/*a*) ratio of 1.211. BZT is metastable in ambient conditions but is reported to have a very high ionic polarization of ≈153 μC cm^−2^.^39,40^ Accordingly, many groups have studied the solid-solutions of BZT with various perovskites in an attempt to utilize the high tetragonality for improved ferroelectric and piezoelectric properties. The addition of BZT was shown to increase the Curie temperature (*T*_C_) of the canonical ferroelectric PbTiO_3_.^41–43^ Some compositions in BZT–BaTiO_3_ system exhibited pseudo-linear dielectric response making them well suited for wide range of temperature-stable dielectric applications.^44–46^ NaNbO_3_ was found to transform from antiferroelectric to a relaxor ferroelectric with a small addition of BZT.^47^

In this work, a solid solution of (1 *− x*)K_0.5_Na_0.5_NbO_3_–*x*BiZn_0.5_Ti_0.5_O_3_ (*x* = 0–0.30) is designed *via* the conventional solid-state method. The effects of BZT doping on the crystal structure and phase transition behavior of KNN ceramic are investigated and the results pertaining to the Rietveld refinement of XRD data, Raman scattering, dielectric and piezoelectric studies are presented.

## 2. Experimental

Polycrystalline samples in (1 − *x*)K_0.5_Na_0.5_NbO_3_–(*x*)BiZn_0.5_Ti_0.5_O_3_ system with *x* = 0, 0.01, 0.02, 0.05, 0.075, 0.10, 0.20 and 0.30 were synthesized using the conventional solid-state reaction route. Stoichiometric amounts of Na_2_CO_3_, K_2_CO_3_, Bi_2_O_3_, TiO_2_, ZnO, and Nb_2_O_5_ (purity > 99.9%) were ball-milled for 12 h. The resultant mixture was dried and calcined at 500 °C for 6 h and then 900 °C for 10 h with intermediate grindings. Subsequently, the calcined powders were uniaxially pressed into pellets of about 1–2 mm in thickness and 10 mm in diameter. The pressed pellets were sintered in air at 1025–1100 °C for 6 h with a heating rate of 3 °C min^−1^ and then furnace cooled to room temperature. All calcinations and sintering steps were carried out in the air.

For confirming the phase purity of calcined powder as well as sintered pellets, X-ray powder diffraction (Bruker-D8 Advance, with CuK_α_ radiation *λ* = 1.54 Å) technique was used. Temperature-dependent X-ray studies were also performed using the same diffractometer equipped with a heated sample stage. Raman scattering measurements were carried out at room temperature using a Raman spectrometer (LabRAM HR800, Horiba Jobin Yvon) in backscattering geometry. The 514.5 nm line of an Ar^+^ laser associated with 20 mW output power was used for excitation. The step-size for the collection of Raman spectroscopy data was 0.526 cm^−1^. A scanning electron microscope (Quanta SEM, FEI) was used for the microstructural analyses.

For dielectric and piezoelectric characterization, polished pellets were coated with a conducting silver paste on either side and cured at 500 °C for 15 min to ensure good contact between the sample surface and silver electrode. The dielectric response of the samples was measured using a Hewlett Packard 4194A impedance analyzer at an applied signal strength of 0.5 V. The measurements were performed as a function of frequency (1 kHz to 1 MHz) and temperature (30 to 450 °C). The temperature was controlled with a programmable oven with an accuracy of ±1 °C. For piezoelectric studies, selected samples were poled in a 100 °C silicon oil bath by applying an electric field of ∼1–1.5 kV mm^−1^ for 1 h. The piezoelectric coefficient *d*_33_ was measured at room temperature using a Piezo-*d*_33_ meter (KCF Tech., Model PM-3001).

## 3. Results and discussion

### 3.1 Structural properties

**Fig. 1:**
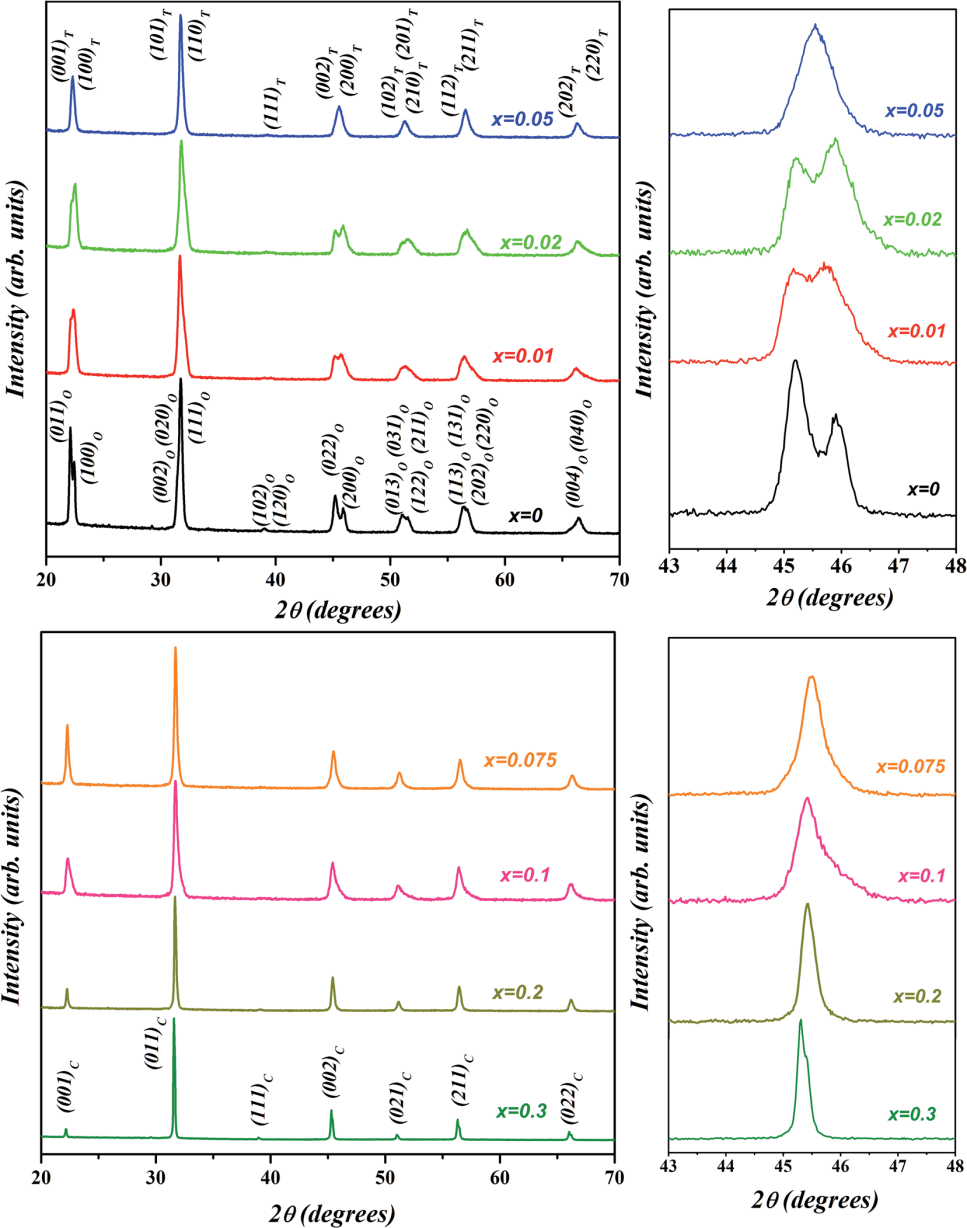
Room temperature X-ray diffraction patterns of (1 − *x*)K_0.5_Na_0.5_NbO_3_–(*x*)BiZn_0.5_Ti_0.5_O_3_ powders for various compositions with 0 ≤ *x* ≤ 0.30.

The room temperature X-ray diffraction patterns of (1 − *x*)K_0.5_Na_0.5_NbO_3_–(*x*)BiZn_0.5_Ti_0.5_O_3_ system for various compositions with 0 ≤ *x* ≤ 0.30 are displayed in Fig. 1. All samples show a pure perovskite structure and no impurity phase is detectable in any of the compositions, confirming that the BiZn_0.5_Ti_0.5_O_3_ has completely diffused into the K_0.5_Na_0.5_NbO_3_ lattice to form a new solid solution in the 0 ≤ *x* ≤ 0.30 range. XRD patterns in the 2*θ* range of 43° to 48° for different compositions are shown in the panels on the right in Fig. 1. A clear splitting and calculated intensity ratio (*I*_022_/*I*_200_ = 1.7) of (022)/(200) peak observed at about 45.5° confirmed the orthorhombic structure for undoped K_0.5_Na_0.5_NbO_3_.^48^ For *x* = 0.01, intensities of 022/200 peak doublet are almost equal; and for *x* = 0.02, there is a reversal in the intensity ratio with *I*_022_/*I*_200_ decreasing to about 0.8. Such reversal in the intensity ratio of peak doublet is characteristic of orthorhombic to tetragonal phase transformation. For a pure tetragonal phase, the intensity ratio of (002)_PC_/(200)_PC_ is expected to be about 0.5 (PC indicates indexing of X-ray diffraction peaks is in pseudo-cubic symmetry).^48^ It can be concluded from the Fig. 1 that the crystal structure of KNN–BZT transforms from the orthorhombic phase (for *x* = 0) to a mixed phase (for *x* = 0.02) with increasing BZT content. For *x* > 0.05, there is increased coalescing of peaks and a gradual weakening of (002)_PC_ peak, indicating the formation of the pseudocubic phase.^49–51^ The asymmetry in the (002)_C_ peak (≈45.4°) in the XRD pattern for *x* = 0.30 sample (Fig. 1) is due to the Cu-K_α2_ radiation.

**Fig. 2:**
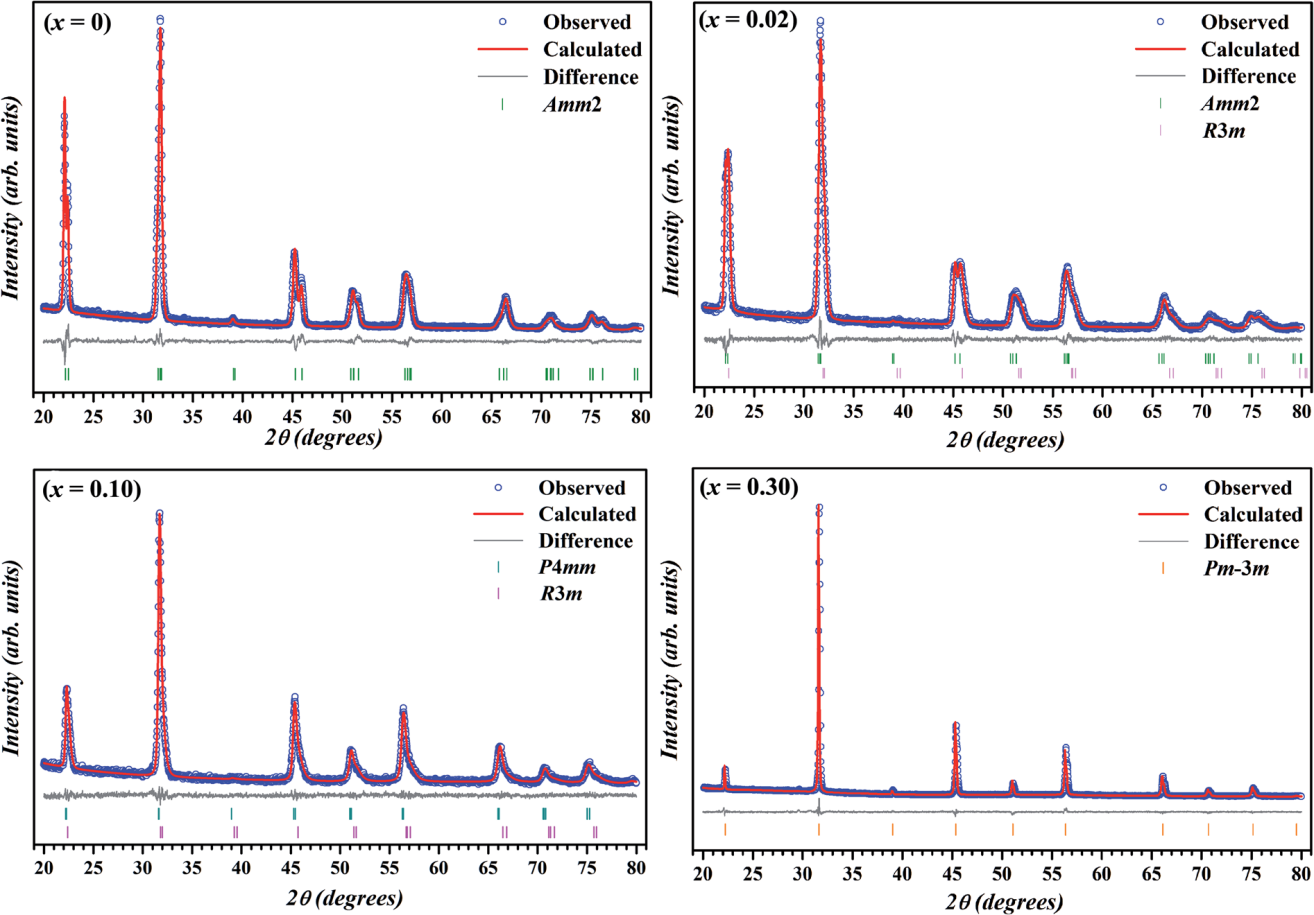
Experimental XRD patterns with Rietveld fit for selected compositions in (1 − *x*)K_0.5_Na_0.5_NbO_3_–(*x*)BiZn_0.5_Ti_0.5_O_3_ system. The experimental data points, calculated pattern and Bragg positions are represented by open circles, solid red line, and vertical ticks, respectively. The bottom gray line is the difference between the experimental and the calculated patterns.

To further study the effect of BZT doping on the crystal structure KNN ceramics, Rietveld refinement of X-ray diffraction was carried out using TOPAS 3.2 software and the calculated patterns (blue circle symbol) along with the observed pattern (solid red line) for the samples with *x* = 0, 0.02, 0.10 and 0.30 are illustrated in Fig. 2. Thin solid gray line and vertical bars at the bottom represent the difference between the calculated and observed patterns and 2*θ* positions of all possible Bragg reflections for selected space group(s), respectively.

During the Rietveld refinement, sample displacement error, unit cell parameters, and background parameters were varied. For the peak shape modeling, a fundamental parameter approach was employed by using the X-ray diffraction data collected on standard alumina (corundum) sample. An orthorhombic space group *Amm*2 with the atomic coordinates reported by Liu *et al.* was used as the starting model for the refinement of XRD data of pure KNN.^52^ During the refinement of X-ray diffraction data of doped samples, Bi and Zn & Ti were assumed to be occupying the A-site and B-site of the perovskite structure, respectively. Further, occupancies of these atoms were fixed according to the nominal stoichiometry for each composition. For doped samples (in the range *x* = 0.01–0.10), refinement using single space group (rhombohedral *R*3*m*, orthorhombic *Amm*2 or tetragonal *P*4*mm*)^51^ resulted in the poor fitting and high *R*-factors. Subsequently, combination of two phases (*Amm*2 + *R*3*m* for *x* = 0.01 & 0.02 and *P*4*mm* + *R*3*m* for *x* = 0.05, 0.075, and 0.10) was used and better fits between observed and calculated pattern were obtained.

The reliability factors and goodness-of-fit indicator obtained from the Rietveld refinement are summarized in Table 1. An excellent agreement between the observed and calculated patterns and low values of the goodness of fit (GOF), *R*_p_, and *R*_wp_ were obtained indicating the reliability of the structural models assigned to each composition. The calculated lattice parameters (*a*, *b*, *c*) with volume (*V*) for various compositions are also listed in Table 1. The effective unit cell volume shows very little change with increasing BZT content. This can be attributed to the competition between the larger average size of octahedral coordinated B-site cations (Zn^2+^ ≈ 0.88 Å and Ti^4+^ ≈ 0.745 Å) and the smaller A-site cation (Bi^3+^ ≈ 1.48 Å) in BZT than in KNN (Nb^5+^ ≈ 0.78 Å and K^+^/Na^+^ ≈ 1.64 Å/1.39 Å). Sutapun *et al.* have reported the existence of rhombohedral phase for the KNN–BZT system for BZT doping level in 0.01 < *x* ≤ 0.03 range.^53^ However, the splitting of (200)_PC_ peak clearly suggests the structure of the composition with *x* = 0.02 to be a combination of orthorhombic and rhombohedral phases. It should be noted that for *R*3*m* space group (111)_PC_ is a doublet whereas (200)_PC_ is a singlet.

**Table 1:** Crystallographic data (space group, lattice parameters) and structure refinement parameters of various compositions in (1 − *x*)K_0.5_Na_0.5_NbO_3_–(*x*)BiZn_0.5_Ti_0.5_O_3_ system

*x*	Crystal system	Space group	Lattice parameters *a* (Å), *b* (Å), *c* (Å), *V* (Å^3^)	*R*_wp_, *R*_p_, GOF
0	Orthorhombic	*Amm*2	*a* = 3.94527(36), *b* = 5.6409(54), *c* = 5.6742(53), *V* = 126.277(47)	3.46, 2.62, 1.08
0.01	Orthorhombic	*Amm*2	*a* = 3.9623(15), *b* = 5.6555(15), *c* = 5.6886(20), *V* = 127.475(20)	6.03, 4.69, 1.26
	Rhombohedral	*R*3*m*	*a* = 5.6067(23), *c* = 6.8027(29), *V* = 185.19(77)	
0.02	Rhombohedral	*R*3*m*	*a* = 5.6066(16), *c* = 6.8095(31), *V* = 184.98(57)	5.53, 4.33, 1.29
	Orthorhombic	*Amm*2	*a* = 3.96909(70), *b* = 5.6592(11), *c* = 5.6833(10), *V* = 127.65(46)	
0.05	Rhombohedral	*R*3*m*	*a* = 5.6021(15), *c* = 6.8130(22), *V* = 185.17(12)	6.36, 4.99, 1.38
	Tetragonal	*P*4*mm*	*a* = 3.97772(28), *c* = 4.0034(34), *V* = 63.342(11)	
0.075	Rhombohedral	*R*3*m*	*a* = 5.6010(30), *c* = 6.8774 (52), *V* = 186.85(15)	5.71, 4.52, 1.20
	Tetragonal	*P*4*mm*	*a* = 3.9781(43), *c* = 3.9927(50), *V* = 63.487(20)	
0.10	Rhombohedral	*R*3*m*	*a* = 5.6207(15), *c* = 6.8313(35), *V* = 186.90(14)	7.61, 6.20, 1.48
	Tetragonal	*P*4*mm*	*a* = 3.9858(40), *c* = 4.00036(84), *V* = 63.554(19)	
0.15	Rhombohedral	*R*3*m*	*a* = 5.64016(67), *c* = 6.9100(14), *V* = 190.368(59)	4.87, 3.98, 1.34
	Cubic	*Pm*3̄*m*	*a* = 3.97895(32), *V* = 62.985(15)	
0.20	Cubic	*Pm*3̄*m*	*a* = 3.99068(43), *V* = 63.554(20)	6.75, 5.16, 1.28
0.30	Cubic	*Pm*3̄*m*	*a* = 3.99453(8), *V* = 63.738(40)	4.78, 3.63, 1.20

**Fig. 3:**
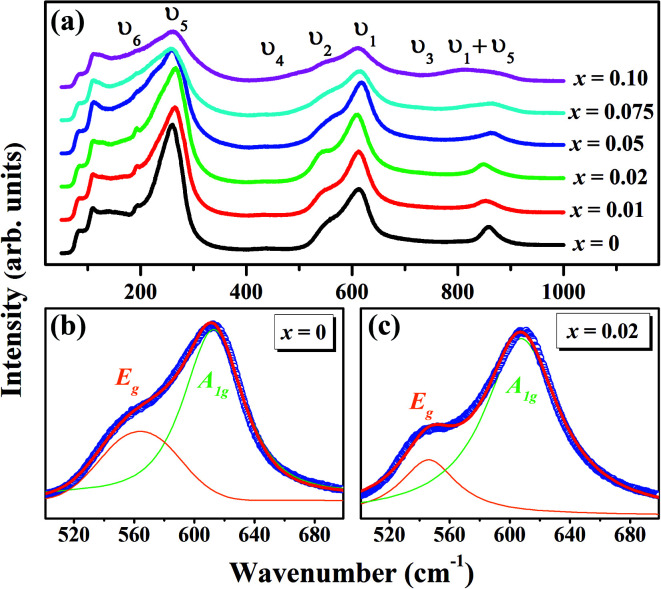
(a) Room temperature Raman spectra of (1 − *x*)K_0.5_Na_0.5_NbO_3_–(*x*)BiZn_0.5_Ti_0.5_O_3_ ceramics with 0 ≤ *x* ≤ 0.10. Fitted Raman spectrum using the sum of two Lorentzian peaks between 500–700 cm^−1^ for (b) *x* = 0 and (c) *x* = 0.02.

The Raman spectroscopy is frequently employed to probe the subtle structural distortions induced both by the tilting of octahedra and by the cationic displacements in perovskites and other related compounds.^54–57^Fig. 3(a) shows the room temperature Raman spectra of (1 − *x*)KNN–(*x*)BZT (*x* = 0.00 to 0.10) samples in the wavenumber range of 50–1000 cm^−1^. The dielectric and Raman studies were performed only for the compositions with *x* ≤ 0.10 as compositions with higher doping exhibited no piezoelectricity and high conductivity at room temperature. Raman peaks of pure KNN (orthorhombic crystal symmetry with *Amm*2 space group) in the region lower than 200 cm^−1^ can be assigned to the translational mode of Na^+^/K^+^, and rotations of NbO_6_ octahedra mode.^56,57^ The weak F_2u_ band observed at 192 cm^−1^ for pure KNN is assigned to the translational modes of A-site cations *versus* NbO_6_ octahedra.^56–58^ With the increase in BZT substitution F_2u_ band further weakens and is not apparent for compositions with *x* ≥ 0.5. The disappearance of the υ_6_ band for high doping suggests that BZT substitution for the A and B site in KNN weakening of NbO_6_ octahedra rotation.

Other internal vibration modes of the NbO_6_ octahedron are,A_1g_(υ_1_) + 1E_g_(υ_2_) + 2F_1u_(υ_3_, υ_4_) + F_2g_(υ_5_) + F_2u_(υ_6_)where 1A_1g_(υ_1_) + 1E_g_(υ_2_) + 1F_1u_(υ_3_) are stretching while rest are bending modes. υ_1_ (at ∼612 cm^−1^ for pure KNN) which represents double degenerate symmetric O–Nb–O stretching vibrations and υ_5_ (at ∼257 cm^−1^ for pure KNN) which represents triple degenerate symmetric O–Nb–O bending vibrations modes are the most prominent bands with relatively strong intensity.^58^ With the increase in BZT content, there is an increase in full-width at half-maximum (FWHM) of the υ_5_ band, especially at higher doping levels. The peak position of υ_5_ band shifts towards higher wavenumber to 267 cm^−1^ for *x* = 0.02 from 257 cm^−1^ for the composition with *x* = 0 and then shifts only slightly towards lower wavenumber on further increase in *x*. Fitted Raman spectra for the deconvolution of A_1g_(υ_1_) and E_g_(υ_2_) band resulting from the stretching modes of the vibrations of the BO_6_ octahedron for *x* = 0 and *x* = 0.02 are shown in Fig. 3(b) and (c). For deconvolution, Raman spectra in the range 450–750 cm^−1^ were fitted as the sum of two Lorentzian peaks. It is seen from the Fig. 3 that υ_2_ band (corresponds to asymmetrical vibration modes of the NbO_6_ octahedra) which appears as a shoulder to the intense υ_1_ band is much more pronounced for the sample with *x* = 0.02 than any other composition under investigation. On increasing the BZT content from 0 to 2%, the wavenumber of υ_2_ decreases from 564 cm^−1^ to 546 cm^−1^ and the wavenumber of υ_1_ decreases from 613 cm^−1^ to 607 cm^−1^. The peak position of F_2g_ (υ_1_ + υ_5_) mode show a similar decrease from 859 cm^−1^ for pure KNN to 853 cm^−1^ for *x* = 0.01 and reaches to the lowest wavenumber (848 cm^−1^) for *x* = 0.02. Incorporation of aliovalent cations at A and B site of KNN induces the variation in the distortion of O–Nb–O angles as well as in the BO_6_ force constant which results in the shift of related modes. Similar behavior of Raman spectra has been observed in other KNN-based solid-solution and confirms the existence of the gradual transition in phase symmetry.^56,58–61^

### 3.2 Microstructure

**Fig. 4:**
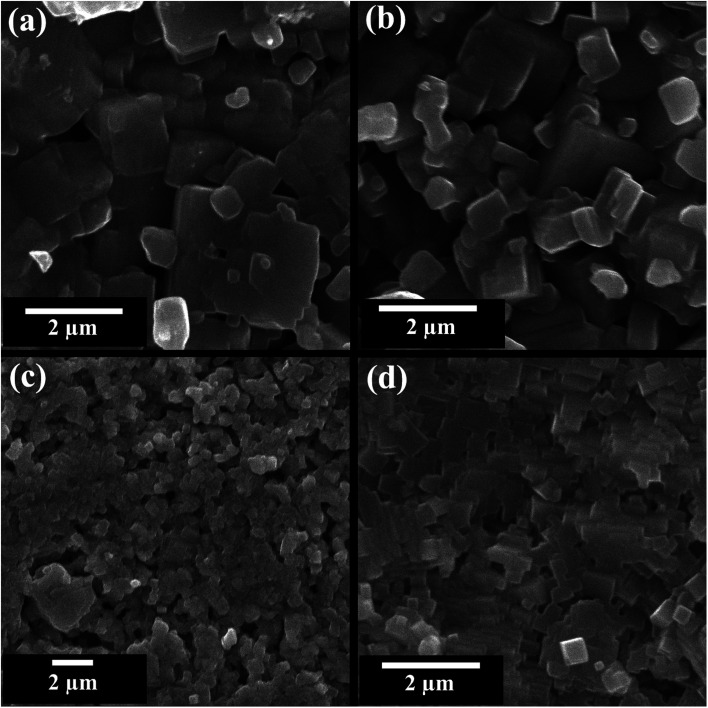
SEM images of the fractured surface of (1 − *x*)K_0.5_Na_0.5_NbO_3_–(*x*)BiZn_0.5_Ti_0.5_O_3_ ceramics for *x* = 0 0.02, 0.05, and 0.10.

Scanning electron microscope (SEM) images of fractured pellet surface of selected compositions (*x* = 0, 0.02, 0.05, and 0.10) in KNN–BZT system are presented in Fig. 4. Micrographs show densely packed grains with a few noticeable pores in all samples. The average grain size as calculated using ImageJ software for pure KNN is 1.06 μm with a large standard deviation of 0.65 μm and it decreases with increasing BZT content to 0.44 ± 0.15 μm for the sample with 10 mol% of BZT content. The relative densities of all samples calculated using theoretical (X-ray) density and sample dimensions are in 89–92% range with a standard error of 2% (Table 2). Another feature of the microstructure of the ceramics under investigation is the noticeable change in the shape of grain with increasing BZT content. At higher BZT doping, grains are cuboid-type in shape with pronounced edges as compared to a more rounded shape for samples with low BZT content.

**Table 2:** Room temperature dielectric constant (*ε*_r_), dielectric loss (tan *δ*), and temperatures of phase transitions (*T*_O–T_ and *T*_T–C_), mean grain size, and the relative density of sintered ceramics for (1 − *x*)K_0.5_Na_0.5_NbO_3_–(*x*)BiZn_0.5_Ti_0.5_O_3_ system

*x*	0	0.01	0.02	0.05	0.075	0.10
*ε*_r_ at 100 kHz	250	483	581	427	410	740
tan *δ* at 100 kHz	0.08	0.74	0.51	0.35	0.26	0.11
*T*_T–C_ (°C)	406	390	383	324	282	264
*T*_O–T_ (°C)	190	187	185	—	—	—
Mean grain size (μm) ± standard deviation	1.06 ± 0.62	1.04 ± 0.70	0.98 ± 0.44	0.72 ± 0.22	0.57 ± 0.20	0.44 ± 0.15
Relative density ± standard error	90 ± 2%	92 ± 2%	91 ± 2%	89 ± 2%	89 ± 2%	92 ± 2%

### 3.3 Dielectric properties

**Fig. 5:**
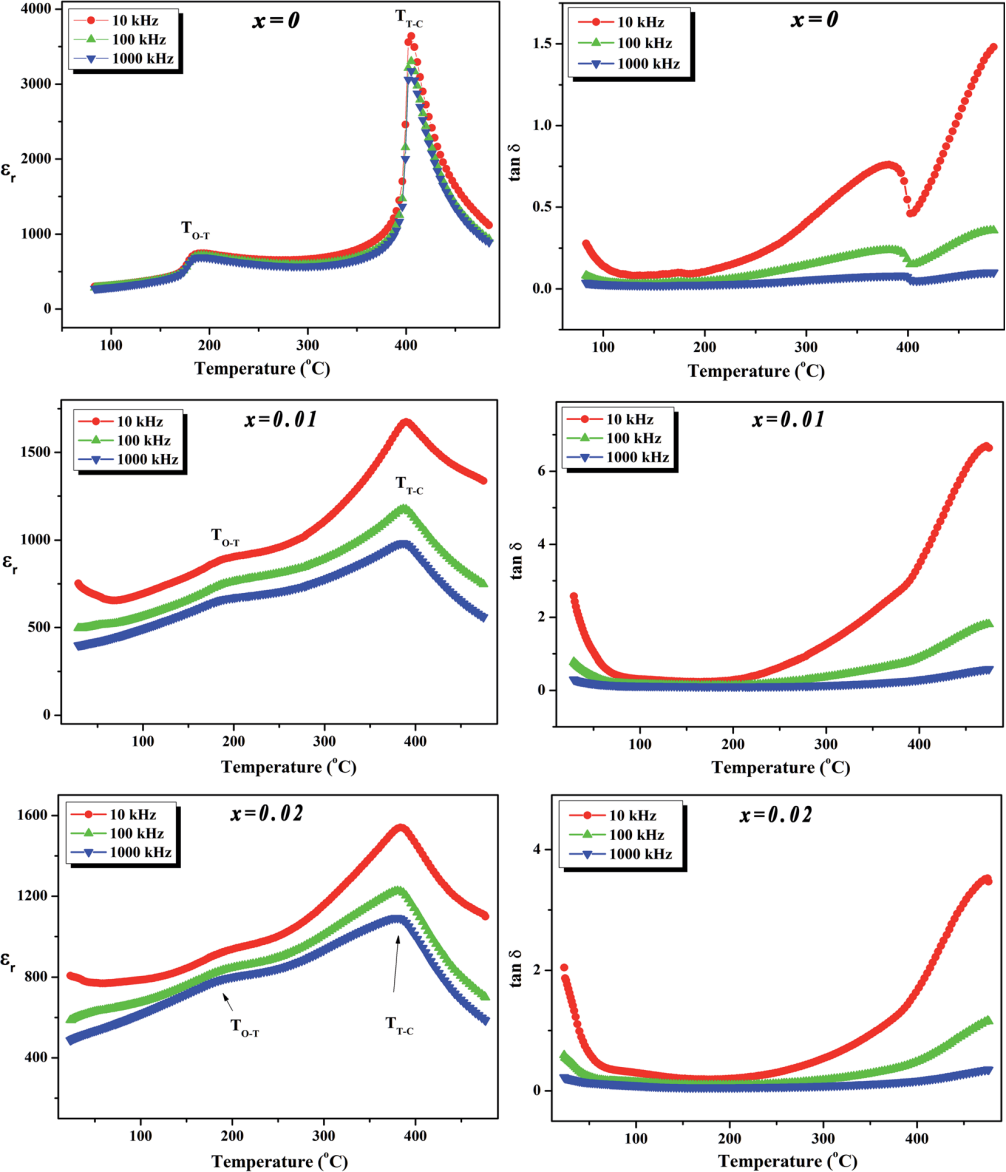
Variation of dielectric constant (*ε*_r_) and dielectric loss (tan *δ*) with temperature at various frequencies for (1 − *x*)K_0.5_Na_0.5_NbO_3_–(*x*)BiZn_0.5_Ti_0.5_O_3_ ceramics in *x* = 0–0.02 range.

**Fig. 6:**
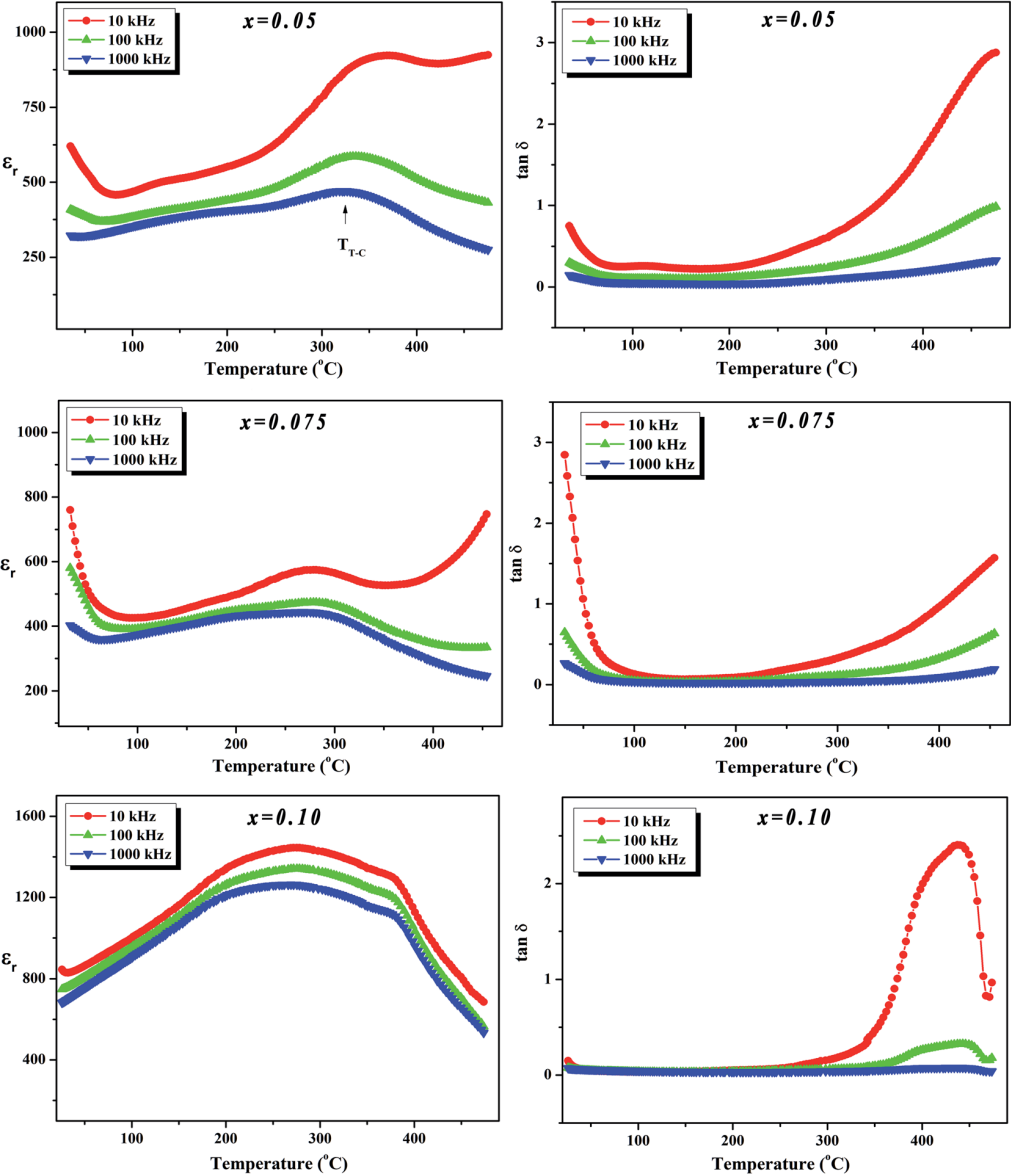
Variation of dielectric constant (*ε*_r_) and dielectric loss (tan *δ*) with temperature at various frequencies for (1 − *x*)K_0.5_Na_0.5_NbO_3_–(*x*)BiZn_0.5_Ti_0.5_O_3_ ceramics in *x* = 0.05–0.10 range.

To further investigate the phase transition of the (100 − *x*)KNN–*x*BZT ceramics, the temperature dependence of relative permittivity and dielectric loss in the temperature range ∼30–450 °C were studied during cooling. The variation of relative permittivity (*ε*_r_) and dielectric loss at three different frequencies (10 kHz, 100 kHz, and 1000 kHz) is shown in Fig. 5 and 6. The values of room temperature dielectric constant (*ε*_r_), dielectric loss (tan *δ*), and the temperatures of phase transitions (*T*_O–T_ and *T*_T–C_) for various composition under investigation are listed in Table 2. Pure KNN sample (Fig. 5) shows two different phase transitions above room temperature: an orthorhombic–tetragonal polymorphic phase transition at *T*_O–T_ ∼ 190 °C and the tetragonal–cubic ferroelectric transition at *T*_T–C_ ∼ 406 °C. It can be seen that after introducing BZT in KNN, the ceramics undergo the tetragonal–orthorhombic and cubic–tetragonal phase transitions, with both *T*_O–T_ and *T*_T–C_ shifted to lower temperatures. The addition of BZT in KNN system induces diffuse phase transition; however, no significant shift in *T*_O–T_ and *T*_T–C_ with the frequency is observed. *T*_T–C_ decreases significantly from 406 °C to 264 °C as *x* increases from 0 to 0.10. The lowering of the temperature of orthorhombic–tetragonal phase transition is comparatively sluggish and *T*_O–T_ decreases from 190 °C for pure KNN (*x* = 0) to 185 °C for *x* = 0.02 sample. Determination of exact *T*_O–T_ was not possible in the sample with *x* ≥ 0.05 due to the increased diffuseness of orthorhombic–tetragonal transition. Another anomaly in *ε*_r_–*T* curve is seen around 380 °C for the *x* = 0.10 composition; however, this anomaly is accompanied with a significant increase in tan *δ* along with a large frequency dispersion at the same temperature suggesting that this feature is related to the defects-related relaxation and not to the structural phase transition.

In the (100 − *x*)KNN–*x*BZT solid solution, the equivalent crystallographic sites of the ABO_3_ perovskite structure are occupied by cations of different valence and sizes (A-site occupied by the Na^+^/K^+^/Bi^3+^ and B-site is occupied by Nb^5+^/Zn^2+^/Ti^4+^) which results in the formation of the random local electric fields owing to the local charge imbalance and localized structural distortions. This composition heterogeneity tends to make the phase transition diffuse instead of sharp as in a normal ferroelectric. This trend is similar to that is observed in the other KNN based solid-solutions.^23,31,48,62,63^ Moreover, such disorders in the system hinder the long-range dipole alignment and result in the lowering of transition temperature. It has been shown that the introduction of smaller A-site cations (such as Li^+^) can induce strong anisotropy *via* frustration between A-site cations of different sizes and stabilize the tetragonal phase.^25^ Addition of Bi_0.50_(K_0.48_Na_0.52_)_0.50_Zr_0.50_Hf_0.50_O_3_ to the Sb-doped KNN results in the formation of R–O–T coexistent phases as well as in R–T phase boundary depending upon the compositions.^32^ Thus, the gradual transformation of room temperature orthorhombic phase to rhombohedral phase and then tetragonal phase in KNN with BZT doping can be ascribed to the Bi^3+^ driven ferroelectricity.

### 3.4 Piezoelectric properties

**Fig. 7:**
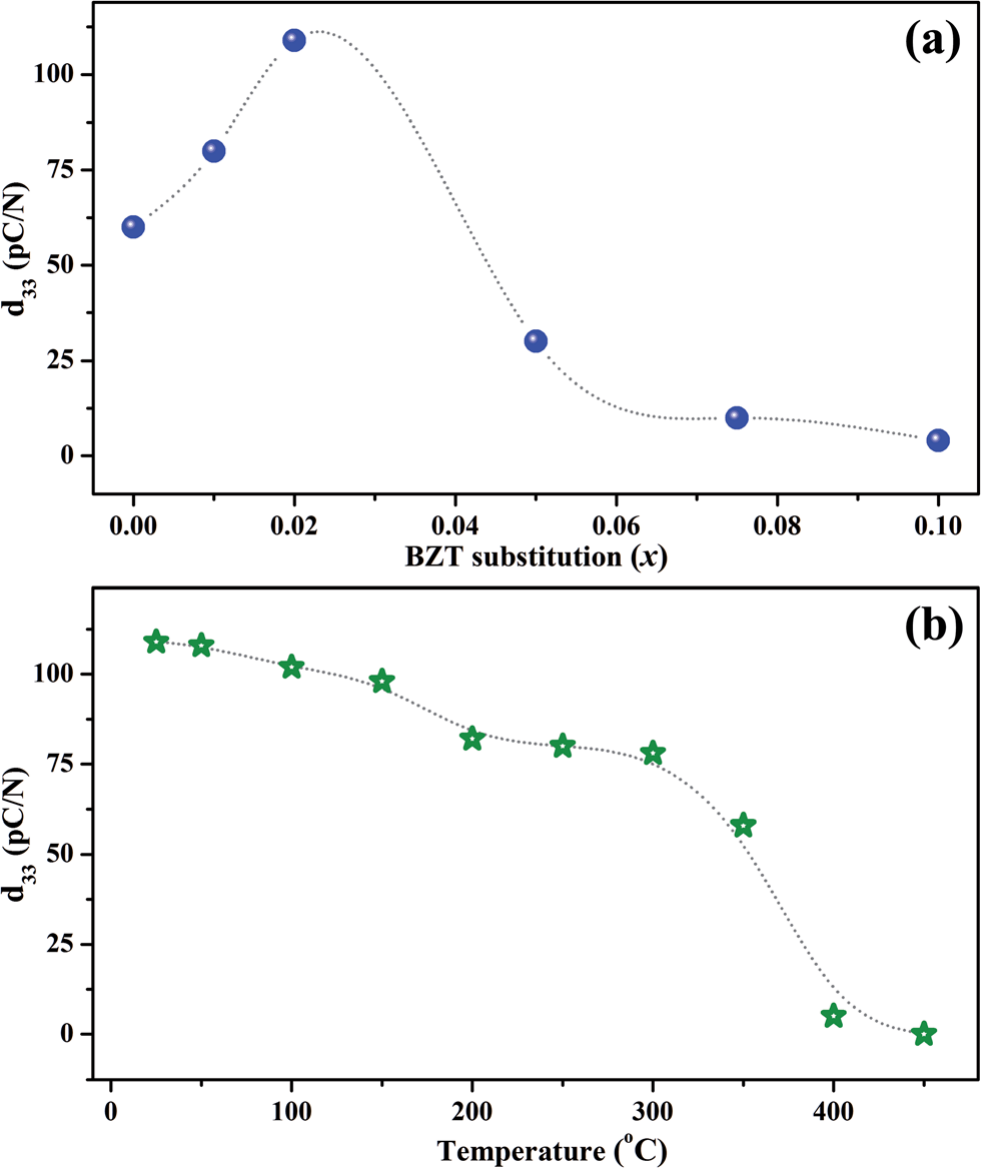
(a) The room temperature piezoelectric coefficient *d*_33_ as a function of BiZn_0.5_Ti_0.5_O_3_ substitution level (*x*). (b) Temperature dependence of the piezoelectric coefficient *d*_33_ for the composition with *x* = 0.02. The dotted lines are a guide to the eye.

The room temperature piezoelectric coefficient *d*_33_ of (1 − *x*)KNN–*x*BZT ceramics as a function of the BZT fraction is shown in Fig. 7(a). The *d*_33_ for pure KNN sample was found to be around 60 pC/N which increased to a maximum of ∼109 pC/N for the composition with *x* = 0.02. The enhancement of piezoelectric coefficient is reported in several KNN-based solid-solutions. The ion substitution is known to greatly improve the piezoelectric properties of such materials through the formation of phase boundaries.^10,13,23,34,62,64–66^ In addition to changes in the crystal symmetry, microstructure induced domain reconfiguration can also improve the piezoelectric properties in optimally-doped piezoelectric materials.^12,17,18,24,26,30,31,64,67,68^ In the present work, superior value of *d*_33_ for the sample with *x* = 0.02 can be attributed to the coexistent orthorhombic and rhombohedral phases at room temperature.

A drastic increase in room temperature conductivity was observed for samples with BZT content (*x*) ≥ 0.05. The value of *d*_33_ decreased to 30 pC/N for the sample with *x* = 0.05 and then decreased further to a mere 4 pC/N for the sample with *x* = 0.10. The leakage current impeded the effective poling of such samples. Also, as demonstrated by X-ray diffraction patterns, the crystal structure of KNN–BZT solid solution gradually changes to the pseudo-cubic type with the increase in BZT concentration. Thus, the poor piezoelectricity exhibited by samples with BZT concentration higher than 2 mol% could be attributed to the structural changes toward pseudo-cubic symmetry and poor poling due to increased leakage current. It must be noted that value of *d*_33_ for pure KNN is reported to be typically in the range of 80–90 pC/N. The lower value of *d*_33_ obtained for pure KNN in the present investigation is possibly related to the processing conditions and lower density.

To check the temperature stability of piezoelectric properties, poled *x* = 0.02 sample was annealed at various temperatures in an oven for 15 minutes. The variation of *d*_33_ with annealing temperature is shown in Fig. 7(b). The value of *d*_33_ shows a sudden drop in the temperature range of 350–400 °C which can be attributed to the change in structure from tetragonal to cubic around *T*_C_. Even though the value of *d*_33_ for the *x* = 0.02 sample at room temperature is low as compared to many of the polymorphic phase boundary compositions based on KNN, sample exhibits a *d*_33_ of 78 pC/N even at 300 °C making it an attractive candidate for high-temperature piezoelectric applications.

### 3.5 High-temperature XRD studies

**Fig. 8:**
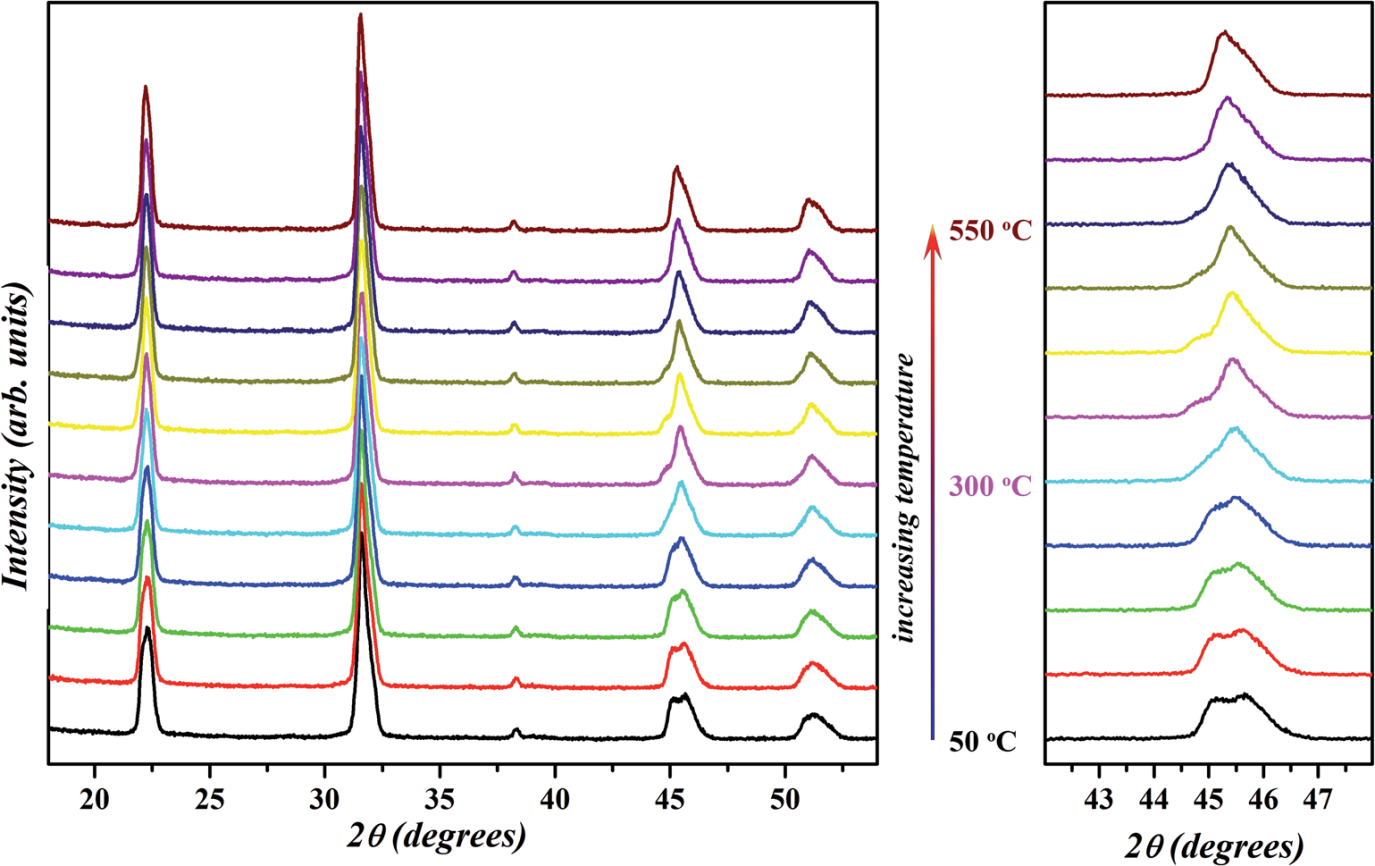
Temperature-dependent X-ray diffraction patterns of *x* = 0.02 sample in 50–550 °C temperature range. Evolution of {200}_PC_ peak with increase in temperature is shown on the right.

Earlier reports on the chemical modification of KNN-based ceramics have demonstrated the realization of a polymorphic phase boundary between orthorhombic and tetragonal phase around room temperature through the decreasing of the temperature of orthorhombic (O) to tetragonal (T) phase transitions.^18,21–26,28,31,35,64,69^ The R–T boundary between rhombohedral (R) and tetragonal (T) phases has also been reported in some systems where the orthorhombic phase in the KNN-based system is suppressed by the introduction of compositional disorder at the crystallographic equivalent sites in perovskite structure.^13–15,20,22,25,37^ Compositions at R–T polymorphic phase boundary in the KNN-based systems attain much better piezoelectric properties than those having R–O or O–T phase boundary.^13^ To further probe the nature of the phase observed for the selected composition *x* = 0.02, temperature dependent XRD was performed in the range of 50–550 °C in a step of 50 °C and the resultant patterns are shown in Fig. 8. The profile of diagnostic (002)_PC_ peak (with 2*θ* ≈ 45.5°) recorded at 50 °C is similar to the one obtained at room temperature (Fig. 1) which suggests that sample is in a mixed orthorhombic–rhombohedral phase. With the increase in temperature, the peak doublet shift towards lower 2*θ* and the intensity of (002)_T_ peak decreases. This is due to the overall expansion of the unit cell and a decrease in the amount of orthorhombic phase. From this, it can be concluded that the structure of KNN–BZT-02 transforms from a mixed orthorhombic–rhombohedral to a mixed rhombohedral–tetragonal and eventually to a cubic phase with the increase in temperature. The observed structural transformation seems to be continuous for the BZT-02 sample in the studied temperature range. Similar behavior has been observed in other KNN-based systems and is usually taken as an indication of the polymorphic phase transition.^18,34,64,70^ Unlike the classic morphotropic phase boundary (MPB) observed in PZT system, orthorhombic–rhombohedral phase coexistence in KNN–BZT shows strong temperature dependence as seen in the Fig. 8.

## 4. Conclusions

In summary, ceramics in KNN–BZT system were successfully fabricated *via* the solid-state reaction method. The room temperature XRD and Raman analyses confirmed the formation of pure perovskite phase for the compositions with *x* ≤ 0.30. Room temperature crystal structure transformed from orthorhombic to cubic through the coexistent tetragonal–rhombohedral phase on BZT substitution. The temperature of orthorhombic–tetragonal, as well as that of tetragonal–cubic transition, decreased with the increase in BZT content. Addition of optimum amount of BZT also improved the piezoelectric properties and sample (1 − *x*)KNN–*x*BZT with *x* = 0.02 showed the piezoelectric coefficient *d*_33_ = 109 pC/N along with a Curie temperature around 383 °C.

## Conflicts of interest

There are no conflicts to declare.

## Supplementary Material
